# A Comparison of Traditional and Geometric Morphometric Techniques for the Study of Basicranial Morphology in Horses: A Case Study of the Araucanian Horse from Colombia

**DOI:** 10.3390/ani10010118

**Published:** 2020-01-10

**Authors:** Pere Miquel Parés-Casanova, Arcesio Salamanca-Carreño, René Alejandro Crosby-Granados, Jannet Bentez-Molano

**Affiliations:** 1 Departament de Ciència Animal, ETSEA, Universitat de Lleida, 25198 Lleida, Spain; 2 Grupo de Investigaciones los Araucos, Facultad de Medicina Veterinaria y Zootecnia, Universidad Cooperativa de Colombia, Arauca 810001, Colombia; asaca_65@yahoo.es (A.S.-C.); rene.crosby@campusucc.edu.co (R.A.C.-G.); jannet.bentez@campusucc.edu.co (J.B.-M.)

**Keywords:** allometry, criollo, phenotypic plasticity, skull, splanchnocranium

## Abstract

**Simple Summary:**

Skull size and shape have been widely used to study domestic animal populations and breeds. Although several techniques have been proposed to quantify cranial form, few attempts have been made to compare the results obtained by different techniques. In this study, two morphometric methods were compared for their ability in describing external morphology. The use of geometric morphometrics combined with multivariate statistical methods is an efficient way to characterize shape and size, thus allowing greater understanding of locally adapted breeds. The Araucanian horse from Colombia inhabits the eastern plains of Arauca. The objective was to compare linear and geometric morphometrics applied to the morphology of the skull of the Araucanian horse, specifically in the basal cranial region. Twenty dry skulls of adult males were examined and were separated into two age groups based on molar eruption and wear. A photograph was taken and a 100 mm scale was placed over each sample. Linear values were obtained from the distance between homologous points from a set of reference points. For the geometric morphometrics analysis, eight paired and five mid-sagittal reference points were used. The geometric morphometric method was more discriminant than linear morphometry and it provides more information about the contour and shape of the face. Future studies should aim to understand the role of phenotypic plasticity in equine race variations and their genetic basis.

**Abstract:**

Skull size and shape have been widely used to study domestic animal populations and breeds. Although several techniques have been proposed to quantify cranial form, few attempts have been made to compare the results obtained by different techniques. While linear morphometrics has traditionally been used in breed characterization, recent advances in geometric morphometrics have created new techniques for specifically quantifying shape and size. The objective of this study was to compare two morphometric methods for their ability to describe external morphology. For this purpose, 20 skull specimens of adult male Araucanian horses were examined. Two age categories were established (the “mature group”, M^3^ not fully erupted to moderately worn, *n* = 7; and the “senile group”, M^3^ totally erupted and highly worn, *n* = 13). Both methods showed that there were statistical differences between generations, but discrimination rates were different between methods with the geometric morphometric analysis obtaining a rate of 97.5%. Although linear morphometrics was found to be compatible with geometric morphometrics, the latter was better able to discriminate the two groups and it also provides more information on shape.

## 1. Introduction

The complex shape of an organism cannot easily be summarized by using only linear measurements (LM) because these measurements are highly correlated with size, and there is no consensus on different size correction methods. Moreover, the homologies of linear distances are difficult to assess with LM, and the same set of distance measurements could be obtained from totally different shapes [1,2].

Skull size and shape have been widely used to study domestic animal populations and breeds [3,4,5]. Although several techniques have been proposed to quantify cranial form, few attempts have been made to compare the results obtained by different techniques. The use of geometric morphometric (GM) techniques is an efficient way to obtain shape features. The landmark-based GM method captures shape information effectively, as it analyses shapes in ways that preserve their integrity and thus avoids collapsing the shapes into a series of linear measurements [6,7]. A generalized Procrustes analysis (GPA) approach eliminates the scale, translational and rotational differences in the coordinate data of the landmarks of subjects. The centroid size (CS) corresponds to the square root of the sum of squared distance between each landmark and the skull centroid [8]. The CS and GPA-scaled coordinates are surrogates for size and shape, respectively. Combining GM methods with multivariate statistical procedures represents a very powerful tool for testing and graphically displaying differences in shape. GM data on horses are scarce. Because of the environment in which they have developed and inhabit, Araucanian horses can be considered as differentiated from other Creole equine breeds [9]. They are adapted to the adverse climatic conditions of the savanna region, used for the extensive management of livestock, for transport and as a working animal [10]. They have a distinct identity that is deeply rooted in the folklore of the region.

GM and LM are very different methods that are used to study morphological variation. The aim of the research was to compare these two techniques applied to the study of the skull morphology of the Araucanian horse, and more specifically to the basicranial part of the skull. This study builds on previous research and aims to contribute to a better understanding of local breeds in the area of Arauca, East Colombia [9,10].

## 2. Materials and Methods

### 2.1. Sampling

We examined skulls from 20 adult male Araucanian horses. This sample was collected from different locations in the Araucanian savannah region during February 2018. Skulls were from animals that had died from unknown causes. Some skulls showed pathological lesions (as assessed by macroscopic examination); however, these lesions did not interfere with our ability to identify the anatomical points of reference. Animals were aged based on molar eruption. Two age categories were established (“mature”, M^3^ not fully erupted to moderately worn, *n* = 7; and “senile”, M^3^ totally erupted and highly worn, *n* = 13).

Image capture was performed with a Nikon® (Nikon Corp., Tokio, Japan) D70 digital camera (image resolution of 2240 × 1488 pixels) equipped with a Nikon AF Nikkor® (Nikon Corp., Tokio, Japan) 28–200 mm telephoto lens. The camera was placed so that the focal axis of the camera was parallel to the horizontal plane and centered on the ventral aspect of the skull. A 100 mm scale was put over each specimen. The software TPSUtil v. 1.50 [11] was used to prepare and organize the digital images. The landmarks were digitized twice using the software TPSDig v. 2.16 [11], by the first author who was also responsible for taking the photographs. The software TPSSmall v. 1.29 [11] showed a high degree of approximation of the shapes in the sample (i.e., it compared the Procrustes to the tangent space distances between individuals) in relation to the reference shape (i.e., tangent space) (*r* = 0.999), which allowed the nature and extent of skull shape changes to be accurately captured in subsequent statistical analyses.

### 2.2. Comparison of Morphometric Methods

In this study, two morphometric methods were compared for their ability to describe external morphology, geometric morphometrics and linear morphometrics.

### 2.3. Geometric Morphometrics

**Figure 1:**
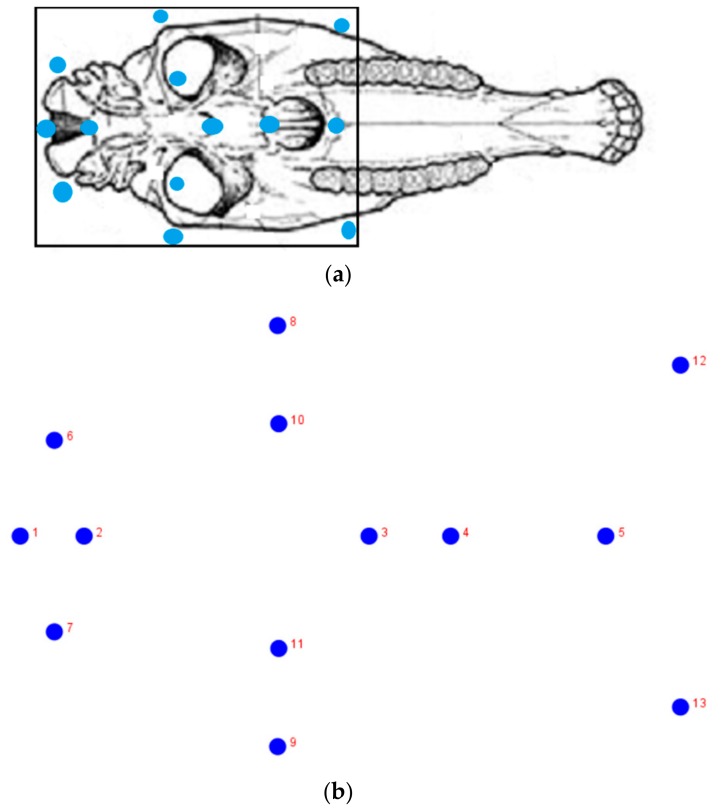
Scheme illustrating the set of 13 landmarks (**b**) used on the ventral aspect of the skull (**a**): 1—Most caudal point of the *foramen magnum*; 2—Most rostral point of the *foramen magnum*; *3*—Most caudal part of the vomer; 4—Most caudal part of the choana; 5—Most cranial part of the choana; 6,7—Two lateral points of *condylus occipitalis*; 8,9—Two lateral points of *tuberculum articulare ossis temporalis*; 10,11—Most medial part of the orbital *pars squamosa ossis temporalis*; 12,13—Most cranio-lateral points of the facial crest.

A GPA to correct differences in position and rotation was used [8] with a total of 8 paired and 5 mid-sagittal landmarks placed on the ventral side of each skull (Figure 1). The landmark coordinates of each specimen were scaled by unit CS. Shape asymmetry was analyzed using configurations superimposed as dependent variables in a Procrustes ANOVA [12], such that the specimen identity was considered as a random effect and the side of the mandible was used as a fixed effect (the effect of the side of the body corresponded to directional asymmetry, the interaction between the side of the body and the specimen identity corresponded to fluctuating asymmetry and the residual term corresponded to the measurement error in the model) [13]. The resampling procedures used 10,000 permutations. To avoid an asymmetrical bias, analyses were performed for the reflected sides relative to the mid-sagittal plane. Overall shape comparison was done with canonical variate analysis (CVA) and a cross-validation discriminant analysis was applied. Finally, a regression of CS (log-transformed values) against shape coordinates was done to assess allometry.

### 2.4. Linear Morphometrics

Linear values were obtained from the distance between homologous points or landmarks of a set of particular landmarks (2–3, 3–5, 6–7, 8–9 and 12–13), according to Driesch’s protocol [14]. The difference between the two groups was assessed by a non-parametric multivariate analysis of variance (NPMANOVA). Group assignment was cross-validated by a leave-one-out cross-validation (jackknifing) procedure.

Analyses were carried out using the PAST v. 2.17c [15] and MorphoJ v. 106c [16] packages. For all tests, statistical significance was demarcated at the 5% level.

## 3. Results

### 3.1. Analysis of Linear Measurements

NPMANOVA revealed significant differences between age groups (*p* < 0.0001). Jackknife discrimination revealed that 5.5% of the specimens were mis-assigned.

### 3.2. Geometric Morphometrics

**Figure 2:**
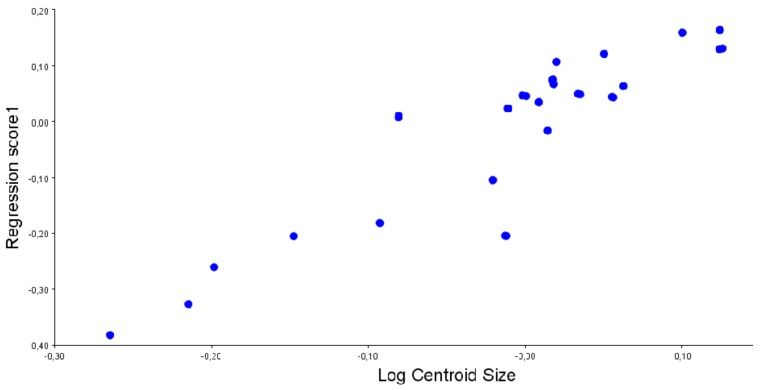
Regression of size (expressed as log of centroid size) versus shape. The analysis demonstrates significant allometry, with 70.8% of the shape variation explained by size. Dimensionless units.

The Procrustes ANOVA for assessing the measurement error showed that the MS for individual variation exceeded the measurement error. Individual variation accounted for 99.9% and 4.5% of the total variation for size and shape, respectively. Error measurement accounted for 0.27% and 0.001% of the total variation for size and shape, respectively. As directional asymmetry was statistically significant (*p* < 0.0001), ulterior analyses were done with reflected sides and averaged individual values. CVA revealed statistical differences between the two groups (*p* < 0.0001), with the discrimination score only failing in 2.5% of cases. Discriminant analyses on the GM dataset containing allometric variation produced the same correct classification rates when compared to the dataset obtained after allometric correction. Regression demonstrated the significant presence of allometry, with 70.8% of the shape variation explained by size. Changes were mainly associated with vomer and palatine bone lengths (Figure 2).

## 4. Discussion

Morphometrics is a tool for the study of form variation. Our results found important differences between landmark and linear-based analyses in terms of the discrimination power. The results show that GM is a more effective method to quantify specific traits in the skull. Moreover, GM can be used to describe developmental pathways and symmetric patterns (not considered here). GM techniques are able to accurately quantify the shape and size of the skull rather than simply provide measurements of width and length, as LM does [17]. In addition, GM has the advantage of visualizing shape changes. Moreover, the cross-validated correct classification rate of the GM dataset containing allometric variation was similar to the correct classification percentages obtained using the non-corrected allometric dataset. This shows that age groups could be discriminated with GM even in the presence of significant allometry.

How to do we reconcile these differences between GM and LM? We suggest that the different results obtained may be due to the difference in measuring only the cranium (as in our study) versus the whole head. The difference may be due to the fact that most studies use absolute, non-size corrected head measurements, whereas here we used “size-free” log-shape ratios to characterize variation in cranial dimensions. It should be noted, however, that different data sets incorporate varying degrees of allometry, which may affect the ability to detect shape differences.

GM also has the advantage of being easy to use, low cost, and quick, and these advantages make it particularly attractive for shared use between research centers. We suggest that shape is more appropriate than size for distinguishing morphologically similar structures, so, differentiation is more informative for breed.

## 5. Conclusions

There were clear differences between the correct classification rates of the two methods. The geometric morphometric analysis provides more information than the linear analysis, and this technique, rather than “conventional” morphometrics, should be considered for the study of domestic breeds. Based on our results, geometric morphometric techniques can potentially contribute in a significant way to the study of locally adapted breeds such as the Araucanian horse.

## 6. Recommendations

Further studies should focus on variations in other planes of the skull, such as the dorsal and lateral plane. Larger sample size is also recommended for future investigations. Developmental stages, from foal to senile animals, should also be considered in order to fully understand the relationship between anatomy and age.
